# Pre‐implant global longitudinal strain as an early sign of pacing‐induced cardiomyopathy in patients with complete atrioventricular block

**DOI:** 10.1111/echo.14942

**Published:** 2021-01-06

**Authors:** Jung Yeon Chin, Ki‐Woon Kang, Sang Hyun Park, Yu Jeong Choi, Kyung Tae Jung, Soyoung Lee, Ho‐Joong Youn

**Affiliations:** ^1^ Cardiovascular Center Division of Cardiology Department of Internal Medicine College of Medicine Eulji University Hospital Eulji University of Korea Daejeon Korea; ^2^ Cardiovascular Center Division of Cardiology Department of Internal Medicine College of Medicine Seoul St. Mary’s Hospital The Catholic University of Korea Seoul Korea

**Keywords:** cardiomyopathy, left ventricular function, myocardial strain

## Abstract

**Introduction:**

Long‐term right ventricular pacing is the only treatment for patients with a complete atrioventricular block (CAVB); however, it frequently triggers ventricular dys‐synchrony with left ventricular (LV) dysfunction. Previous studies showed that an early decline of LV global longitudinal strain (GLS) predicts pacing‐induced LV dysfunction. We aimed to investigate the potential ability of the initial LV strain to predict pacing‐induced cardiomyopathy (PICM) through long‐term follow‐ups.

**Methods:**

We retrospectively enrolled 80 patients with CAVB with normal LV function who were implanted with dual‐chamber pacemakers between 2008 and 2018. Echocardiographic data and parameters (including longitudinal, radial, and circumferential strain based on speckle‐tracking) were analyzed for the pre‐implant (≤6 months) and post‐implant periods. PICM was defined as a ≥10% reduction in the left ventricular ejection fraction (LVEF) resulting in an LVEF of <50% during the post‐implant period. Predictors of PICM were identified using Cox proportional hazard models.

**Results:**

Patients who developed PICM were more likely to exhibit lower baseline LV GLS, as well as wider native and pacing QRS durations, than those who did not develop PICM (*P* = .016, *P* = .011, and *P* = .026, respectively). In the multivariate analysis, pre‐implant LV GLS (hazard ratio: 1.27; 95% confidence interval 1.009–1.492; *P* = .004) was independently associated with the development of PICM.

**Conclusion:**

A lower baseline LV GLS predicts an increased risk of PICM. Patients with CAVB exhibiting low GLS are at increased risk of PICM. More frequent follow‐up visits are warranted in these patients, who may also require de novo His‐bundle pacing or an upgrade to biventricular pacing.

## 1 INTRODUCTION

Since 1958, long‐term right ventricular (RV) apical pacing has been used to treat patients with symptomatic bradycardia. However, this method is less effective than native human pacing, given that it can only produce approximately one quarter of the normal conduction velocity and may induce heterogeneous electrical ventricular activation.^1^ RV apical pacing results in ventricular dys‐synchrony and deterioration of left ventricular (LV) function, similar to a left bundle branch block.^2^ Therefore, patients with pacemakers are at increased risk of atrial fibrillation, heart failure (HF), and death. HF caused by chronic and frequent RV pacing is defined as pacing‐induced cardiomyopathy (PICM). Patients with PICM require an additional procedure such as RV septal pacing or an upgrade to biventricular pacing.^1^, ^3^, ^4^, ^5^, ^6^, ^7^, ^8^, ^9^, ^10^

Despite this knowledge, predicting PICM remains challenging.^11^, ^12^, ^13^, ^14^ One single‐center retrospective study reported that male sex and a wide native QRS duration were independent predictors of PICM.^14^ Another study demonstrated that global longitudinal strain (GLS) in the LV 1 month after pacemaker implantation accurately predicted pacing‐induced LV dysfunction at 1 year.^15^ LV GLS is known as a more powerful indicator of LV function than the LV ejection fraction (LVEF) in patients with HF, acute myocardial infarction, and valvular heart disease.^16^ LV strain also exhibits superior prognostic ability for major adverse cardiac events when compared with LVEF. Although a previous study reported that LVEF is not a predictor of PICM,^14^ no studies have investigated whether LV strain prior to pacemaker implantation can predict PICM. Therefore, in the present study, we aimed to investigate the potential ability of LV strain (including GLS) to predict PICM.

## 2 METHODS

### 2.1 Study population

We retrospectively reviewed data from 131 patients who received a pacemaker due to a complete atrioventricular block (CAVB) between January 2008 and January 2018. Inclusion criteria were as follows: baseline LVEF ≥ 50%, implantation of a single‐chamber ventricular or dual‐chamber pacemaker (but not of an implantable cardioverter‐defibrillator or biventricular pacemaker), echocardiography ≤6 months before and after pacemaker implantation in our hospital using the same manufacturer's echocardiography machine. Patients with atrial fibrillation, congenital heart disease, more than a moderate degree of valvular heart disease, a history of open‐heart surgery, and a history of myocardial infarction were excluded. Patients were also excluded due to technical issues related to echocardiographic image analysis if the examinations were performed at another hospital, the echocardiographic images could not be analyzed due to low frame rate, there was a poor echo window, or the images were in a non‐DICOM format. PICM was defined as a ≥10% decrease in LVEF, resulting in an LVEF < 50%. Patients with other definite causes of cardiomyopathy including atrial fibrillation on follow‐up ECG, those with high right atrial (RA) episodes, and those with >1% automatic mode switching in the pacemaker program were also excluded. The study protocol was approved by the Institutional Review Board of Eulji University Hospital (IRB No. EMC 2018‐01‐013‐006).

### 2.2 Echocardiographic analyses

Patients underwent echocardiography (Vivid 7; GE Vingmed) with a 3.5‐MHz phased‐array transducer. A total of two consecutive cycles in three parasternal short axis views (mitral, papillary, and apical) and three longitudinal standard apical views (apical four‐, two‐, and three‐chamber views) were obtained. The resulting loops were digitally stored and subsequently analyzed offline using EchoPAC workstation 7.1.1 software (GE Vingmed). The LVEF was calculated using the modified Simpson's method. Speckles were tracked frame by frame throughout the LV myocardium over the course of one cardiac cycle, following which the basal, mid, and apical regions of interest were defined. Thereafter, each image was carefully inspected, and the segments that failed to track were manually adjusted. If more than one segment could not be tracked, there was a lack of a full cardiac cycle, or there was significant foreshortening of the left ventricle, then the measurements were considered unreliable, and the patient was excluded from our study. Global radial and circumferential strain values were calculated as the average strain across the parasternal views. GLS was calculated as the average strain across the apical four‐, two‐, and three‐chamber views. Analyses were performed on single, optimal cardiac cycle. All analyses were conducted offline, and the analyzer was blinded to the identities of the patients and other clinical data.

### 2.3 Inter‐ and intra‐observer variability

To assess inter‐observer variability in measurements of global strain, a subset of 10 randomly selected echocardiographic images (19% of patients) were assessed by two observers (Jung Yeon Chin and Ho‐joong Yoon) who were blinded to each other's results. Four weeks later, these measurements were reviewed by the same investigator, who remained blinded to the previous results. Both inter‐ and intra‐observer variability were expressed in terms of intra‐class correlation coefficients and percent variability.

### 2.4 Statistical analyses

Continuous variables are expressed as medians and interquartile ranges, while categorical variables are expressed as percentages. Continuous and categorical variables were compared using Student's *t* tests and Pearson's χ^2^ tests, for which *P*‐values < .05 were considered statistically significant. The survival curve for PICM was generated using the Kaplan–Meier method. Univariate and multivariate predictors of PICM were analyzed using Cox proportional hazard models. The variables used for the univariate analysis included sex, age (per 1‐year increase), coronary artery disease, hypertension, diabetes, beta‐blocker use, calcium channel blocker use, diuretic use, initial echocardiographic parameters (including left atrial anterior–posterior diameter, LVEF, LV longitudinal strain, LV radial strain, and LV circumferential strain), initial ECG findings (including native and paced QRS duration), and ventricular pacing percentage. Receiver operating characteristic curve analysis was performed to determine the optimal sensitivity and specificity of LV global longitudinal peak systolic strain. Values higher than the cutoff values were considered positive. All data were analyzed using SPSS (version 20.0; SPSS Inc).

## 3 RESULTS

**Figure 1:**
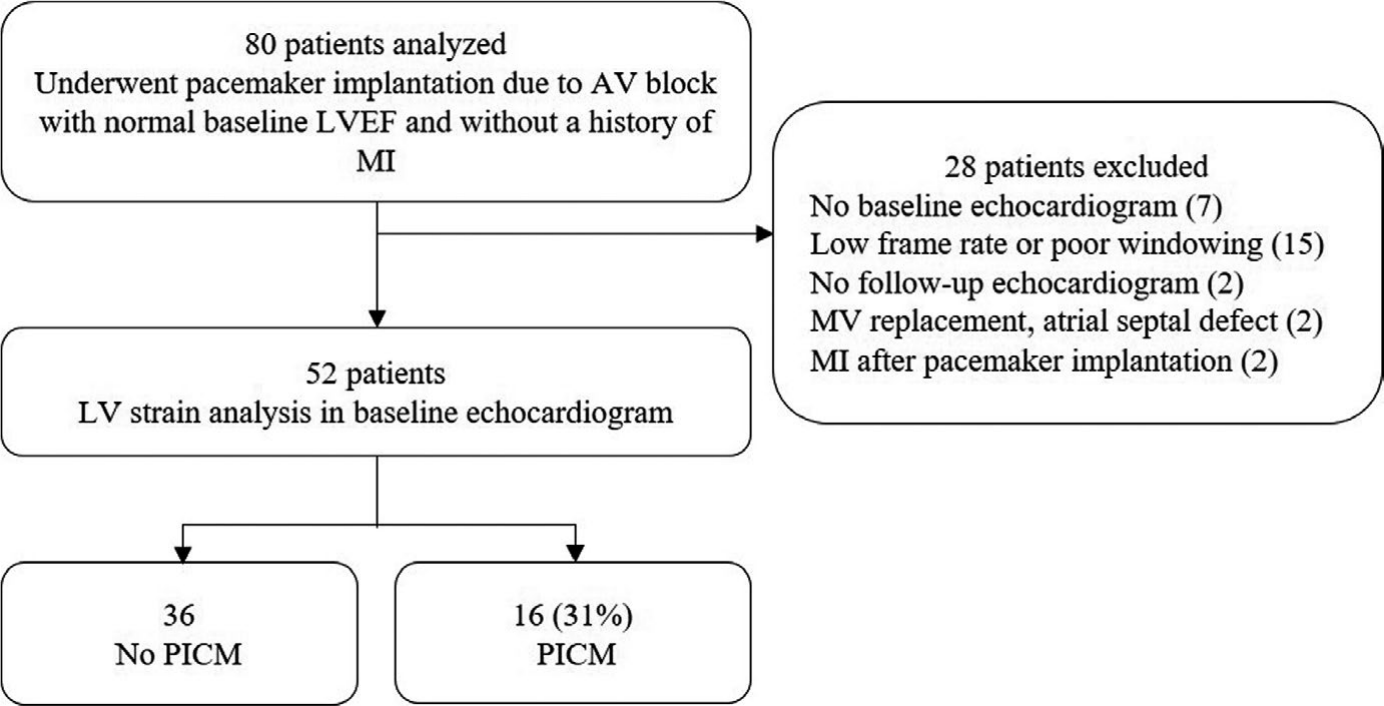
Patient flowchart after pacemaker implantation. AV = atrioventricular; LVEF = left ventricular ejection fraction; MI = myocardial infarct; MV = mitral valve; PICM = pacing‐induced cardiomyopathy

We initially enrolled 80 patients who received a pacemaker due to a CAVB with normal baseline LVEF values and no atrial fibrillation on initial and follow‐up ECG. We further excluded 28 patients for various reasons: Seven patients were excluded because they did not undergo baseline echocardiography at Daejeon Eulji University Hospital, 15 were excluded due to poor image quality (low frame rate/poor windowing), two were excluded because they did not undergo follow‐up echocardiography, one was excluded due to previous mitral valve replacement, one was excluded due to an atrial septal defect with pulmonary stenosis, and two were excluded due to myocardial infarcts after receiving a pacemaker. Of the 52 remaining patients, 16 (31%) were diagnosed with PICM, while the remaining 36 were not (69%). The mean follow‐up duration among these 52 patients was 5.2 years (Figure 1).

The baseline demographic characteristics of the included patients are presented in Table 1. There were no significant differences in age, sex, or underlying disease between the two groups. However, patients in the PICM group were more likely to use ACE inhibitors, angiotensin II receptor blockers (ARBs), and diuretics than those in the non‐PICM group.

**Table 1:** Baseline characteristics of the included patients stratified by pacing‐induced cardiomyopathy occurrence

	Total (n = 52)	No pacing‐induced cardiomyopathy (n = 36)	Pacing‐induced cardiomyopathy (n = 16)	*P*
Demographic characteristics, comorbidities, and medications				
Age, year	78 (72.3–84.0)	78 (72.2–83.0)	79 (65.3–89.3)	.858
Female sex, %	34 (65.4%)	25 (69.4%)	9 (56.3%)	.922
Hypertension, %	25 (48.1%)	15 (41.7%)	10 (62.5%)	.169
Diabetes, %	11 (21.2%)	8 (22.2%)	3 (18.8%)	.779
Coronary artery disease, %	7 (13.5%)	4 (11.1%)	3 (18.8%)	.461
ACE inhibitor or ARB, %	20 (38.5%)	10 (27.8%)	10 (62.5%)	.019
Beta‐blocker, %	6 (11.5%)	3 (8.3%)	3 (18.8%)	.283
CCB, %	7 (13.5%)	4 (11.1%)	3 (18.8%)	.461
Diuretics, %	13 (25.0%)	6 (16.7%)	7 (43.8%)	.039
Hemoglobin, g/mL	12.3 (11.0–13.6)	12.3 (11.9–13.9)	12.2 (11.0–13.5)	.859
Creatinine, mg/dL	0.80 (0.60–1.00)	0.78 (0.60–0.98)	0.90 (0.50–1.10)	.918

Note

Data are presented as the median and interquartile range.

Abbreviations: ACE = angiotensin‐converting enzyme; ARB = angiotensin receptor blocker; CCB = calcium channel blocker.

**Figure 2:**
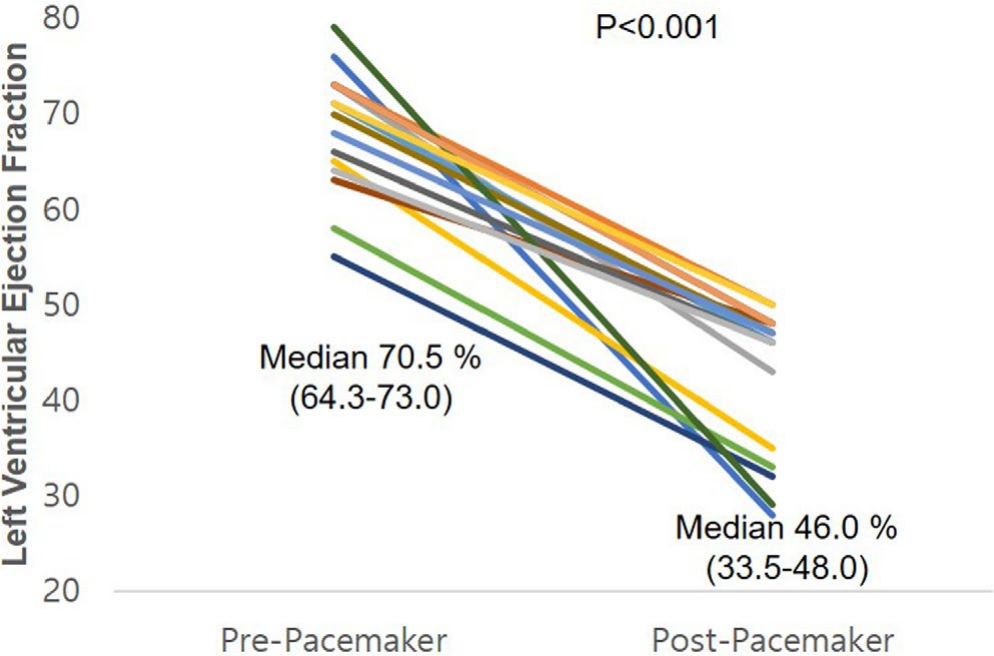
Left ventricular ejection fraction decreased from 70.5% (interquartile range 64.3–73.0) to 46.0% (interquartile range 33.5–48.0) in patients with pacing‐induced cardiomyopathy

**Figure 3:**
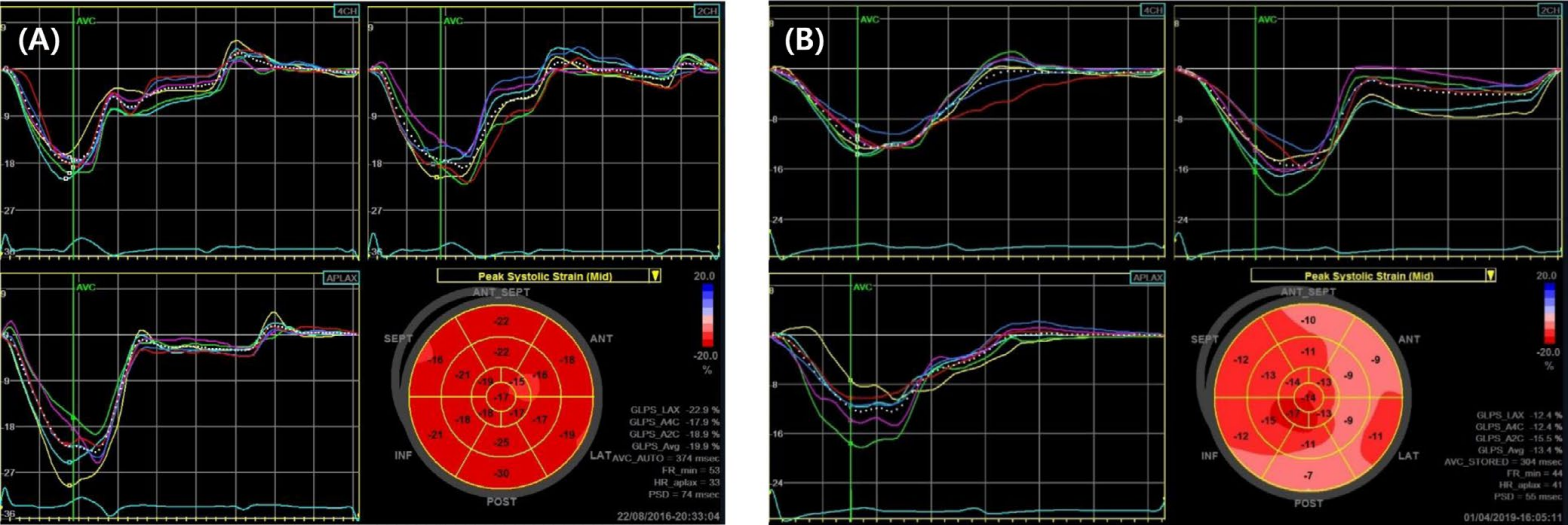
Two representative cases of initial LV GLS values. The initial LV GLS of patient (A) without PICM was −19.9%, while the LVEF was 65%. The initial LV GLS of patient (B) with PICM was −12.4%, although the initial LVEF was 64%. LVEF = left ventricular ejection fraction; LV GLS = left ventricular global longitudinal strain; PICM = pacing‐induced cardiomyopathy

The initial LVEF was similar in both groups (70.5% [61.7–77.8%] vs 70.5% [64.3–73.0%], *P* = .922). However, post‐implantation LVEF values were significantly lower in the PICM group than in the non‐PICM group (46.0% [33.5–48.0] vs 62.0% [58.0–67.5], *P* < .001; Figure 2). Global circumferential and radial strain values were similar in both groups. However, the initial GLS was significantly higher in patients without PICM than in those with PICM (*P* = .016; Table 2). A representative example of the difference in the initial GLS between patients with and without PICM is shown in Figure 3, even though the initial LVEF values were nearly identical.

**Table 2:** Baseline characteristics of the included patients stratified by pacing‐induced cardiomyopathy occurrence

	Total (n = 52)	No pacing‐induced cardiomyopathy (n = 36)	Pacing‐induced cardiomyopathy (n = 16)	*P*
Echocardiographic parameters				
LA diameter, mm	38.0 (35.0–42.0)	38.0 (34.0–41.0)	39.5 (36.3–44.8)	.177
Pre‐implant LVEF, %	70.5 (63.9–76.0)	70.5 (61.7–77.8)	70.5 (64.3–73.0)	.922
Post‐implant LVEF, %	58.5 (48.0–64.8)	62.0 (58.0–67.5)	46.0 (33.5–48.0)	<.001
Global longitudinal strain, %	−20.1 (−22.8 to −17.0)	−21.2 (−23.7 to −18.2)	−18.0 (−20.6 to −14.5)	.016
Global circumferential strain, %	−21.2 (−25.5 to −15.5)	−25.7(−22.0 to −18.2)	−19.2 (−22.2 to −13.1)	.110
Global radial strain, %	47.9 (33.9–61.9)	48.2 (42.0–61.0)	39.8 (26.8–65.8)	.289
Electrophysiologic parameters				
Heart rate, bpm	60.0 (43.0–66.0)	54.5 (39.8–64.8)	60.0 (54.0–76.0)	.126
Native QRS duration, ms	134 (100–154)	124 (92–146)	150 (118–162)	.011
Pacing QRS duration, ms	151 (128–162)	103 (147–159)	153 (141–177)	.026
Non‐apical pacing, n (%)	12 (23.1%)	11 (30.6%)	1 (6.3%)	.057
Ventricular pacing percentage	98.0 (74.3–99.0)	96.0 (66.5–99.0)	99.0 (80.0–99.0)	.513

Note

Data are presented as the median and interquartile range.

Abbreviations: LA = left atrium; LVEF = left ventricular ejection fraction.

**Figure 4:**
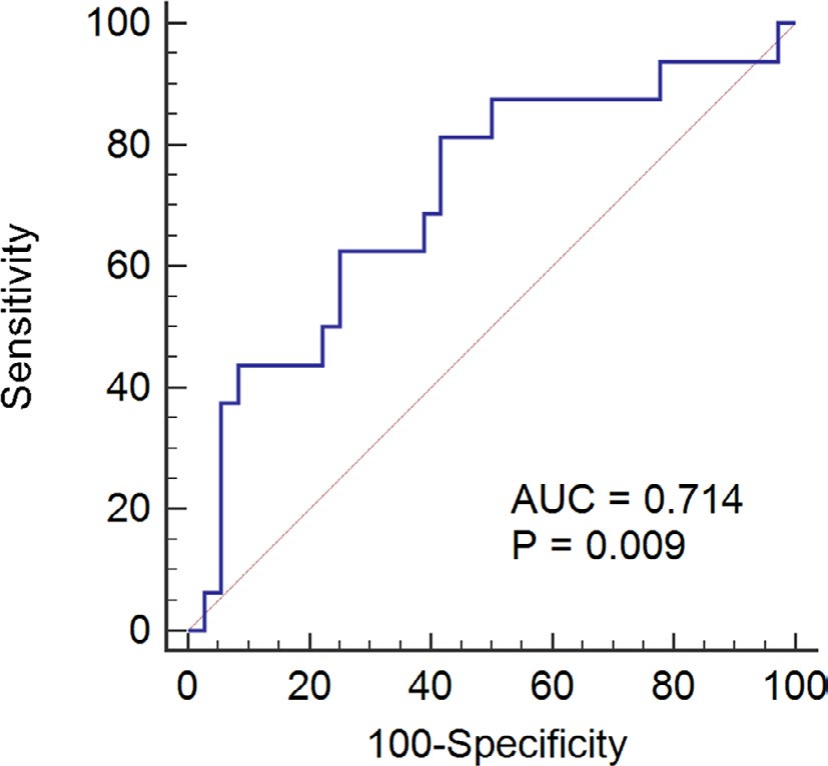
With a global longitudinal peak systolic strain (GLPSS) cutoff value of <−20.7, PICM could be predicted with a sensitivity of 81.3% and a specificity of 58.0%. PICM = pacing‐induced cardiomyopathy

**Figure 5:**
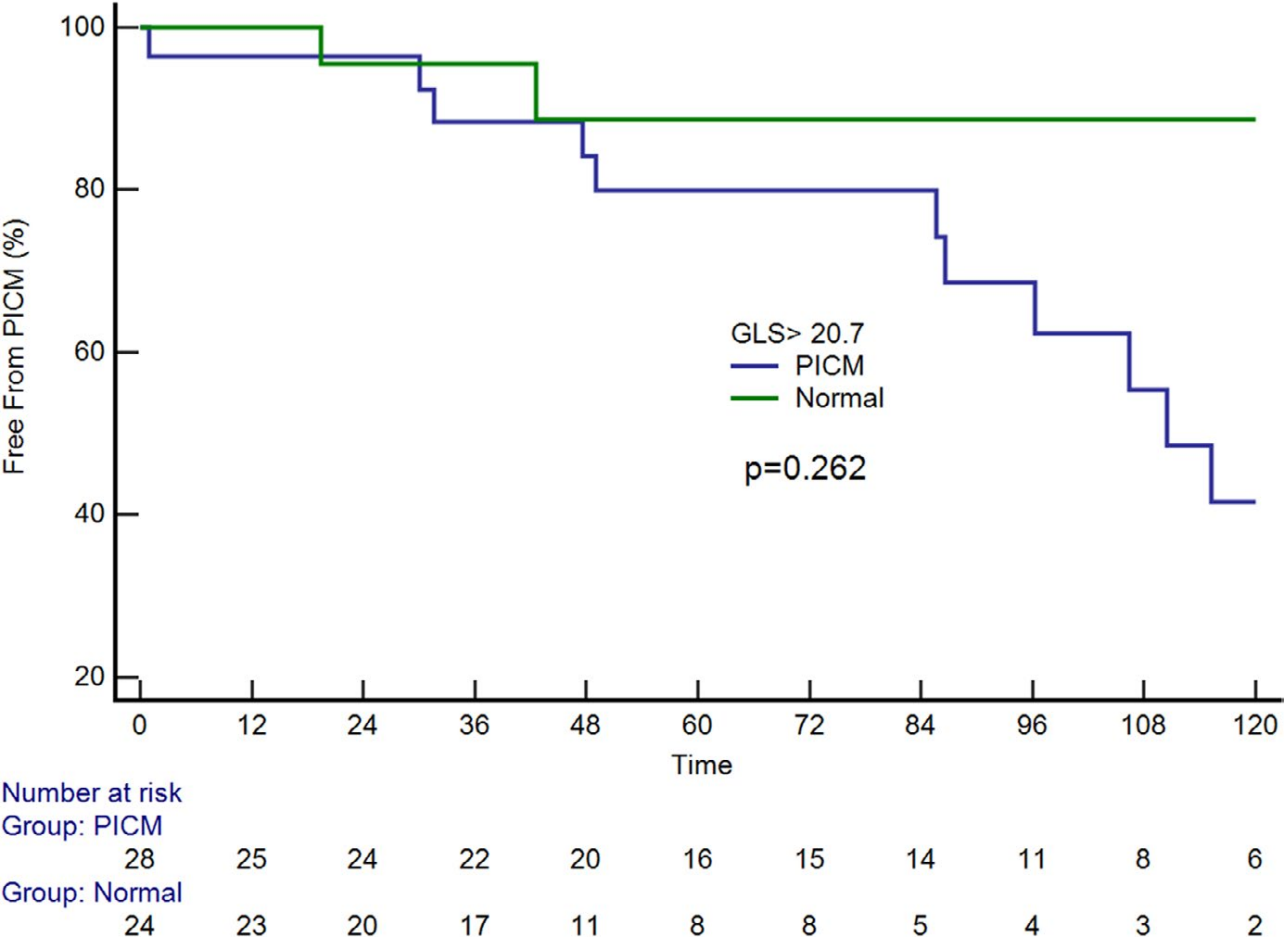
Kaplan–Meier curve showed no significant difference between the two groups. However, pacing‐induced cardiomyopathy tended to occur more frequently in patients with a global longitudinal strain <−20.7%

Native and pacing QRS durations were longer in patients with PICM than in patients without PICM (Table 2). In the univariate Cox analysis, the use of diuretics, LV GLS, and the native and pacing QRS durations were identified as predictors of PICM development. Multivariate analysis revealed that LV GLS (hazard ratio: 1.27; 95% confidence interval: 1.009–1.492; *P* = .04) and native QRS duration (hazard ratio: 1.031; 95% confidence interval: 1.003–1.059; *P* = .030) remained independently associated with the development of PICM (Table 3). For LV GLS, a cutoff value of <−20.7 predicted PICM, with a sensitivity of 81.3% and a specificity of 58.0% (Figure 4). Our analysis indicated that LV GLS exhibited a greater predictive probability for the development of PICM. Kaplan–Meier analysis revealed no significant differences between the two patient groups (*P* = .219; Figure 5). However, PICM occurred relatively more frequently in patients with an LV GLS <−20.7 than in those with an LV GLS greater than the −20.7 threshold.

**Table 3:** Factors predicting the development of pacing‐induced cardiomyopathy

Factor	Univariate			Multivariate		
	Hazard ratio	95% CI	*P*	Hazard ratio	95% CI	*P*
Female sex	0.574	1.027–1.550	.193			
Age (per 1‐y increase)	0.715	0.944–1.046	.867			
Coronary artery disease	0.461	0.362–9.421	.461			
Hypertension	2.330	0.696–7.823	.170			
Diabetes	0.808	0.184–3.552	.777			
Beta‐blocker use	2.538	0.453–12.236	.290			
Calcium channel blocker use	1.846	0.362–9.421	.461			
Diuretic use	3.889	1.038–14.566	.044			
LA anterior–posterior diameter	1.057	0.974–1.147	.185			
LVEF (per 1% increase)	0.997	0.931–1.066	.920			
LV longitudinal strain	1.218	1.028–1.443	.023	1.27	1.009–1.492	.004
LV radial strain	0.980	0.944–1.017	.283			
LV circumferential strain	1.111	0.975–1.266	.114			
Native QRS duration	1.029	1.005–1.053	.016	1.031	1.003–1.059	.030
Paced QRS duration	1.028	1.002–1.054	.037			
Ventricular pacing percentage	1.010	0.981–1.040	.506			

Abbreviations: CI = confidence interval; LA = left atrial; LV = left ventricular; LVEF = left ventricular ejection fraction.

### 3.1 Inter‐ and intra‐observer variability

The inter‐observer variability for global strain was low: Intra‐class correlation coefficients were 0.87 (95% confidence interval: 0.45–0.97, *P* = .004), 0.80 (95% confidence interval: 0.04–0.96, *P* = .023), and 0.74 (95% confidence interval: −0.01 to 0.94, *P* = .032) for global longitudinal, radial, and circumferential strain, respectively. Percent variability for global longitudinal, radial, and circumferential strain was 6.2%, 10.0%, and 11.7%, respectively. The intra‐observer variability for global strain was also low, with intra‐class correlation coefficients of 0.93 (95% confidence interval: 0.75–0.98, *P* = .001), 0.94 (95% confidence interval: 0.72–0.96, *P* = .001), and 0.91 (95% confidence interval: 0.64–0.98, *P* = .001) for global longitudinal, radial, and circumferential strain, respectively. Similarly, percent variability for each type of strain was 2.2%, 6.4%, and 4.9%, respectively.

## 4 DISCUSSION

The present study aimed to investigate the ability of LV strain to predict PICM. Our findings demonstrated that initial GLS was significantly associated with the development of PICM. In particular, GLS values <−20.7 had a sensitivity of 81% and specificity of 58% for predicting PICM.

Previous studies have reported incidence rates for PICM ranging from 9% to 26%.^11^, ^12^, ^13^, ^14^ The PICM rate may have been higher in our study (31%) because we included a homogenous group of patients with CAVB that exhibited nearly 90% ventricular pacing. In the Mode Selection in Sinus Node Dysfunction (MOST) trial, patients with >50% ventricular pacing had an increased probability of HF, hospitalization, or death.^17^ Our hospital's follow‐up policy may also explain the higher rate of PICM in our study. Nearly all patients who receive a pacemaker at our hospital have frequent follow‐up visits and undergo echocardiography once per year and pacemaker programming every 6 months. With these frequent visits, we are able to identify patients with PICM before they exhibit symptoms of HF, while some hospitals wait until patients exhibit symptoms before performing such examinations.

In our study, initial rates of diuretic, ACE inhibitor, and/or ARB use were higher in the PICM group than in the non‐PICM group—a potential limitation of our study. These patients may have experienced dyspnea or edema in the past because these medications are treatments for HF, which may in turn indicate that they were at increased risk of developing PICM.

In accordance with previous findings, our data indicate that PICM may be more common in patients with relatively longer native QRS durations. A previous study reported that a wide native QRS duration was associated with higher rates of death, heart transplantation, and LV device implantation in patients with non‐ischemic cardiomyopathy and ventricular tachycardia.^14^ In a randomized trial comparing patients with biventricular and RV‐only pacing, the benefit of biventricular pacing seemed to be greater in patients with a native QRS duration > 110 ms, although the interaction did not reach statistical significance.^18^ In our previously published study, we demonstrated that pacing QRS duration is a major determinant of PICM.^19^ Although native QRS duration was identified as a significant predictor of PICM (hazard ratio = 1.031; *P* = .030) in the current study, pacing QRS duration exhibited significant predictive ability in the univariate analysis only, showing a positive trend in the multivariate analysis. Given that the previous study relied on data from a different population (ie, three regional hospitals), additional large‐scale multi‐center cohort studies are required to determine whether pacing or native QRS duration can predict PICM occurrence.

A previous meta‐analysis reported that LV GLS exhibited greater prognostic value for global LV dysfunction than LVEF in patients with HF, acute myocardial infarction, and valvular heart disease.^16^ Strain values are free of angle dependency and reflect the motion of the subendocardium. LV GLS can be used to detect subclinical LV impairment, especially when the LVEF is normal or near normal. In other diseases such as myocardial infarction, valvular heart disease, and end‐stage renal disease, LV GLS has been identified as a more sensitive method of determining LV function than LVEF.

There are three potential explanations for the increased risk of PICM in patients with GLS values <−21%. This value is considered high when compared to a GLS value of >−18%, which has been considered normal. However, a recent meta‐analysis of 2396 individuals with a mean age of 42 years conducted from 2011 to 2018 indicates that the new normal value for GLS is −21% (range: −22.7% to −19.2%). This new range coincides with findings we observed in healthy children, whose mean LV GLS is −20.2%. The discrepancy between our results and the standard normal value may be due to our use of the latest data when analyzing GLS value. Although GLS is dependent on weight, age, and load, variations among equipment vendors may also explain the observed differences in the cutoff values. According to the abovementioned meta‐analysis, the normal range for GLS varied among vendors, with values for Tomtec and General Electric being significantly higher than those for Toshiba, Philips, and Siemens. In our study, data were obtained from a General Electric Echopac device, which may again explain the higher value. Finally, cumulative ventricular pacing percentage (>80%) was higher, while the duration of follow‐up was longer (4.5 years), in our study than in previous studies. This high pacing burden results in more instances of PICM because premature ventricular contractions activate vagal afferents and alter autonomic tone. This finding suggests that patients with supernormal LV GLS values can be protected from PICM.

Patients in the PAVD study exhibited low GLS values 1 month after receiving a pacemaker. GLS values at 1 month also predicted pacing‐induced LV dysfunction at 12 months (area under the curve = 0.80, optimal GLS threshold: <14.5, sensitivity: 82%, specificity: 75%). However, there were no significant differences in baseline GLS between patients with and without LVEF decline (−16.3 vs −17.5). Most patients included in this study had a second‐degree atrioventricular block (82%), rather than a third‐degree block, the latter of which requires more pacing. In the PAVD study, mean cumulative ventricular pacing at 12 months was only 53.5%, whereas it was 80% in our study.

### 4.1 Study strengths

Our study is the first to analyze baseline LV strain as a prognostic indicator of PICM. In addition, we utilized recent real‐world data and analyzed LV strain using the same instrument. In contrast to previous studies regarding PICM, we selected patients with CAVB to exclude bias related to the percentage of pacing. Patients with CAVB are mostly pacing‐dependent, whereas the percentage of pacing is diverse among patients with sick sinus syndrome or tachy–bradycardia syndrome. Furthermore, we carefully excluded patients with other causes of cardiomyopathy.

### 4.2 Study limitations

Our study was clearly limited by the small number of patients and its single‐center design. Furthermore, inclusion bias may exist because we excluded patients with poor echocardiographic images, patients who did not undergo follow‐up echocardiography, and patients who underwent echocardiography at other hospitals. However, due to the follow‐up policy at our hospital, we are potentially able to identify patients with PICM slightly earlier than other hospitals.

## 5 CONCLUSION

Our study provides evidence that the prognostic value of GLS for predicting adverse cardiac events is superior to that of LVEF. Indeed, our findings indicate the PICM may begin to develop even when LV function is still normal, and that the risk of PICM can be predicted based on baseline GLS. As such, patients with low GLS are at increased risk of developing PICM. These patients warrant closer follow‐up examinations and must be considered for de novo His‐bundle pacing or an upgrade to cardiac resynchronization therapy to avoid LV dys‐synchrony.

## ACKNOWLEDGMENTS

Dr. Ki‐Woon Kang contributed to the conceptualization to this work and we appreciated it.

## CONFLICT OF INTEREST

None declared.

## Data Availability

Data available on request from the authors.
